# Embryonic response to long-term exposure of the marine crustacean *Nephrops norvegicus* to ocean acidification and elevated temperature

**DOI:** 10.1002/ece3.860

**Published:** 2013-11-15

**Authors:** Hannah K Styf, Helen Nilsson Sköld, Susanne P Eriksson

**Affiliations:** The Department of Biological and Environmental Sciences – Kristineberg, University of GothenburgKristineberg 566, SE-451 78, Fiskebäckskil, Sweden

**Keywords:** Carbon dioxide, cardiac performance, climate change, crustacean, development rate, hypercapnia, Norway lobster, ocean acidification, oxidative stress, rate of oxygen consumption

## Abstract

Due to anthropogenic CO_2_ emissions, our oceans have gradually become warmer and more acidic. To better understand the consequences of this, there is a need for long-term (months) and multistressor experiments. Earlier research demonstrates that the effects of global climate change are specific to species and life stages. We exposed berried Norway lobsters (*Nephrops norvegicus*), during 4 months to the combination of six ecologically relevant temperatures (5–18°C) and reduced pH (by 0.4 units). Embryonic responses were investigated by quantifying proxies for development rate and fitness including: % yolk consumption, mean heart rate, rate of oxygen consumption, and oxidative stress. We found no interactions between temperature and pH, and reduced pH only affected the level of oxidative stress significantly, with a higher level of oxidative stress in the controls. Increased temperature and % yolk consumed had positive effects on all parameters except on oxidative stress, which did not change in response to temperature. There was a difference in development rate between the ranges of 5–10°C (*Q*_10_: 5.4) and 10–18°C (*Q*_10_: 2.9), implicating a thermal break point at 10°C or below. No thermal limit to a further increased development rate was found. The insensitivity of *N. norvegicus* embryos to low pH might be explained by adaptation to a pH-reduced external habitat and/or internal hypercapnia during incubation. Our results thus indicate that this species would benefit from global warming and be able to withstand the predicted decrease in ocean pH in the next century during their earliest life stages. However, future studies need to combine low pH and elevated temperature treatments with hypoxia as hypoxic events are frequently and increasingly occurring in the habitat of benthic species.

## Introduction

As a consequence of the anthropogenic carbon dioxide (CO_2_) emissions, our oceans have gradually become warmer and more acidic. According to IPCC (2007), observations since 1961 have revealed that over 80% of the heat added to the climate system has been absorbed by the oceans. Surface temperatures are estimated to increase by approximately 4°C by 2100 (A1Fl scenario, IPCC 2007. For the last 25 million years, the acidifying effect of CO_2_, a process named ocean acidification (OA) has been buffered by carbonate ions (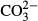), keeping pH between 8.0 and 8.3 (Widdicombe and Spicer 2008). However, since mid-18th, this buffering capacity has been challenged by the rate of CO_2_ uptake, and ocean pH has decreased by 0.1 units. In a “business-as-usual” regime, the current mean pH of 8.1 is projected to drop by another 0.4 units by the end of century (Caldeira and Wickett 2003, 2005). This implies a three times more acidic oceanic environment. How these future environmental changes will affect marine organisms is of great concern.

Previous research suggests that effects of elevated temperature and OA will be specific to species and life stages. So far, most studies have focused on juveniles and adults. As early life stages may be particularly sensitive to stress and thereby impose a bottleneck in species success, effects on, for example, embryos and larvae must be thoroughly investigated (Byrne 2011). There is also a demand for long-term (months), multistressor experiments, more comparable to reality than the majority of studies to date, which are short (acute) to medium term (weeks) with a single stressor. Long-term experiments could also verify preceding results in the field of OA and climate change (Whiteley 2011). Previous experiments with combined exposure to OA and elevated temperature have found interactive effects with, for example, decreased thermal tolerance under elevated Pco_2_ (Metzger et al. 2007; Walther et al. 2009). As calcification was believed to be the most sensitive to a decreased oceanic pH, many studies have focused on calcifying species (Orr et al. 2005; Fabry et al. 2008). However, not all calcifying organisms are necessarily at risk (Ries et al. 2009; Findlay et al. 2011). Crustaceans have been found to compensate and maintain or even increase their rate of calcification during short-term exposure to acidified water (Spicer et al. 2007; Whiteley 2011). Over longer periods of time (weeks–months), this may infer a higher energy expenditure and/or diversion of energy from other key biological aspects (Wood et al. 2008; Stumpp et al. 2011; Whiteley 2011). Hence, responses in essential physiological processes, other than calcification, to climate change and OA are likewise important to study in crustaceans.

The decapod crustacean, *Nephrops norvegicus* is found on the continental shelf and slope throughout the northeastern Atlantic Ocean and the Mediterranean Sea (Barquín et al. 1998; Johnson et al. 2013). Global catch is approximately 70,000 tonnes year^−1^, making it commercially the most important crustacean in Europe (Nofima 2012). It lives a relatively stationary life, digging burrows in soft, muddy substrata, from a few meters, down to 800 m depth and is a key species in these ecosystems (Johnson et al. 2013). The seabed is naturally reduced with oxygen penetrating the sediment by only a few millimeters, and the adult *N. norvegicus* is adapted to cope with this challenging environment to a certain point (Eriksson et al. 2013). Embryonic development occurs externally with the eggs attached to the female pleopods where they are subjected to the ambient condition of the sediment burrow. The developing embryo relies on its yolk for energy and hatch as planktotrophic larvae after about 4–13 months. The incubation period in *N. norvegicus* is positively correlated with latitude (Powell and Eriksson 2013). Earlier studies have shown that the incubation period decreases with elevated temperature in the range of 8–15°C (Dunthorn 1967). The maximum temperature, above which development rate no longer can increase, is not known. Furthermore, it is not known whether the increased development rate have associated costs. Suboptimal temperatures and/or pH levels may cause stress to the embryos and result in a higher metabolic demand. This could lead to a depletion of energy stores before onset of hatching and thereby compromise the hatching process itself and potentially also species fitness, ecosystems, geographical distribution etc. (Pörtner and Farrell 2008; Riebesell et al. 2010).

As patterns in cardiac performance and rate of oxygen consumption reflect metabolic rate and may reveal critical environmental conditions, measurements of these parameters are highly useful (Frederich and Pörtner 2000). During early crustacean development, heart rate equals cardiac output (Harper and Reiber 2004). Furthermore, metabolism can produce potent reactive oxygen species (ROS), which cause oxidative stress if the production of ROS exceeds antioxidant capacity (Hamdoun and Epel 2007). Oxidized proteins aggregate into protein carbonyls, which commonly accumulate in tissues with age and due to environmental perturbation and are thus frequently used as biomarker for stress (Dalle-Donne et al. 2003; Almroth et al. 2005).

The objective of this study was to evaluate crustacean embryonic tolerance in a near future environment and to obtain new insights into crustacean embryonic development. This was achieved by investigating long-term effects of combined exposure to a range of temperatures (5–18°C) and two pH levels (current vs. −0.4 units), on the embryonic development of *N. norvegicus*. We examined embryonic responses by quantifying proxies for development rate and fitness including: % yolk consumption, mean heart rate, rate of oxygen consumption, and oxidative stress. Our hypothesis was that, reduced pH would have an impact on *N. norvegicus* embryos and that the effect would be potentiated by elevated water temperature. The results were expected to assist when predicting recruitment and future importance of *N. norvegicus,* in the muddy substrata ecosystem.

## Material and Methods

### Collection of specimens

Berried female *N. norvegicus* were caught in Gullmarsfjorden on the west coast of Sweden (N58°15′W11°25′), using lobster pots, and then transferred to a 100-l tank with flow-through untreated seawater at Sven Lovén Centre for Marine Sciences, Kristineberg. Conditions in the field during collection (September 2010) were 33 PSU and T 14°C.

### Experimental design

The experiment was conducted between October 2010 and February 2011, with monthly measurements, that is, in mid-November, mid-December, mid-January, and mid-February. The lobsters were kept individually in 15-L plastic tanks supplied with temperature and pH-set water from a header tank system (Hernroth et al. 2012). Aquaria where supplied with artificial burrows (PVC-pipes) serving as refuge. No sediment was added to avoid confounding effects from sediment-associated substances. The females were kept in a 12 h:12 h light:dark regime and fed once a week with shrimp (*Pandalus borealis*) of 5 g wet weight.

Seventy-two females (i.e., 72 egg clutches) were included in 12 combinations of six temperatures (5, 10, 12, 14, 16, and 18°C) and two pH levels (control/field pH and −0.4 pH-units). Each egg clutch was considered as a true replicate (*n* = 6 egg clutches per combination). As higher temperatures infer increased development rate, experimental end points differed between treatment groups. Fifty-two percentage of all clutches began hatching within the experimental period. However, 86% of all clutches reached the last embryonic stage. Due to the size and complexity of the experiment, the temperature treatments had to be divided into four different temperature-constant rooms, in order to maintain a minimum difference between the water (WT) and air (AT) temperatures: (1) WT & AT 5°C, (2) WT 10 & 12°C, AT 11°C, (3) WT 14 & 16°C, AT 15°C, and (4) WT & AT 18°C. Water flow was kept at 4 L h^−1^ which was high enough to prevent the AT deviation of 1 degree to have an impact on the WT of the treatments. The temperature span in the experiment constituted >90% of the water temperatures recorded at 32 m depth during July 2009–July 2012 in Gullmarfjorden (Weather Station, Sven Lovén Centre, Fiskebäckskil, Sweden). The mean field temperature during JULY 01, 2007 to JULY 01, 2012 was 10.1°C ± 4.1 (SD). Less than 5% of the field measurements were below 5°C, and less than 1% of the field measurements were above 18°C.

### Seawater temperature and chemistry

The animals were acclimatized from a field temperature of 14°C (at collection), to their target experimental temperature over 13 days prior to the experiment. At the end of adult temperature acclimation, pH was lowered in the “low pH header tanks” by pH-computers (AB Aqua Medic probes, Bissendorf, Germany), bubbling bottled gas (100% CO_2_) into the tanks. The pH levels were controlled in the header tanks in order to stabilize the system. Oxygen concentration, temperature (WTW oxi 340i with an OxiCell 325 oxygen- electrode, Weilheim, Germany), and salinity (WTW microprocessor conductivity meter, LF 196) were measured on a daily basis.

Furthermore, total alkalinity (Eppendorf BioPhotometer, Hamburg, Germany), temperature, and pH (WTW pH 3310 with a SenTix 41 electrode, Weilheim, Germany) were measured twice a week. Total alkalinity was measured following Sarazin et al. 1999;. pH_total scale_ was determined using TRIS-(2-amino-2hydroxy-1.3 propanediol) and AMP-(2-aminopyridine) buffers. Pco_2_ (μatm) was calculated using the CO_2_sys software. Dissociation constants from Mehrbach et al. 1973 were used and refitted by Dickson and Millero 1987. Salinity of incoming seawater during the experiment was 32.0 ± 0.04 (mean ± SE) PSU.

### Development

A minimum of ten eggs were photographed at each sampling using a Leica stereomicroscope (MZ16 A, Leica Microsystems, Wetzlar, Germany). The embryonic development in *N. norvegicus* has been described previously by dividing the development into categorical stages of nonlinear duration by visual assessment of characters, such as first occurrence of eye pigmentation and chromatophores (Powell and Eriksson 2013). We found this categorical measurement of nonlinear character of age to be crude in relation to the other parameters quantified in our study. Instead a nonsubjective and comparable calculation of yolk size was developed as a continuous measurement of embryonic age to assess developmental rate and to obtain a measure in relation to the other physiological parameters. This was carried out by calculating the percentage of pixels covering yolk in the collected pictures with a sagittal view of the embryo (*n* = 5), using the graphic software, ImageJ. Development rate for eggs at each temperature and pH was calculated by averaging the decrease in % yolk day^−1^ for each group. At each sampling, the eggs were also classified as alive or dead.

### Cardiac performance

Five eggs were taken from each female and transferred under-water in the experimental seawater into a flat bottom flask (63 mL). The embryonic heart rate was measured under a stereomicroscope (40×) in the experimental rooms, where air temperature did not deviate more than ± 1°C from the flask/experimental water (see above). The number of heartbeats (BPM = beats min^−1^) was counted during 1 min for each egg (either manually or at >200 BPM from film in slow motion). Only eggs with a regularly BPM (Eriksson et al. 2006) during the whole minute of measurement were used in the analysis and compared with the developmental stage.

### Rate of oxygen consumption

Rate of oxygen consumption (nmol O_2_ egg^−1^ h^−1^) of the egg was measured as a proxy for embryonic metabolic rate; by optical oxygen microsensors (NTH Pst1, PreSens, Regensburg, Germany) consisting of 140-μm silica optic fibers and sensor tips <50 μm, mounted in needle-type housing. Optodes were calibrated in air-saturated seawater (100%) and in oxygen-free seawater, using sodium disulfide (0%). Two different transmitters were used, a microx TX3 fiber–optic transmitter and an Oxy-4 micro transmitter with the manufacturer's software (PreSens). Two 1-l beakers were filled with filtered water (45 μm), aerated, and pH-adjusted to control pH (8.1) and low pH (−0.4 units) by CO_2_-bubbling the low pH-water. Ten eggs from each female were collected and placed in glass vials (1.634 ± 0.004 mL). The eggs were washed three times with filtered seawater of the experimental pH to minimize occurrence of bacteria and other microorganisms. Empty control vials were included in each temperature set to subtract background respiration from the egg respiration values. Between runs, the vials were cleaned with EtOH (96%), distilled water (MilliQ, Billerica, MA) and left to dry. The vials containing washed eggs were filled with 100% air-saturated water and sealed with a silicone/Teflon cap. The measurements were then taken in a water bath set at the target temperature. Start values of oxygen concentration was recorded for a period of 5–10 min with the microsensors measuring every 5 sec. End-values were recorded in the same manner, after 2–23 h of incubation depending on temperature treatment. To avoid oxygen gradients during shorter incubations, the vials were left in the water bath and turned every 5 min. During longer incubations (overnight), the vials were put in zip bags and placed in a header tank where the vials were under constant movement.

### Oxidative stress (protein carbonyls)

Protein Assay Reagent Kit were purchased from Pierce, Thermo Scientific. All other chemicals were from Sigma-Aldrich (St Louis, MO).

Oxidative stress (nmol protein carbonyls mg protein^−1^) was measured via a reaction with 2,4-dinitrophenylhydrazine (DNPH), followed by trichloroacetic acid (TCA) precipitation, as previously described (Levine et al. 1990; Reznick and Packer 1994; Yan et al. 1996). Ten eggs (including yolk and embryo protein) were homogenized in ice-cold 50 mM phosphate buffer (pH 7.4), containing 0.1% (w/v) digitonin and protease inhibitor cocktail (P8340). The homogenates were centrifuged at 10^4^ g for 20 min at 4°C. Supernatant samples of 1 mg protein were incubated with 10 mM DNPH in 2 M HCl for 2 h and then precipitated with TCA on ice before being centrifuged at 10^4^ g for 10 min at 4°C. Pellets were washed three times with ethanol–ethyl acetate (1:1) followed by centrifugation in order to remove free DNPH and lipid contaminants. After resolubilizing the pellets in guanidine hydrochloride solution at pH 2.3 by incubation at 37°C, absorbance was read at 360 nm using a plate reader. Negative controls not subjected to DNPH were run in parallel, and these values were subtracted from the DNPH-treated samples. Finally, total protein concentration was measured using the BCA Protein Assay Reagent Kit, and data were expressed as nmol protein carbonyls mg protein^−1^ using the molar absorption coefficient of 22,000 M^−1^ cm^−1^ for DNPH derivatives.

### Data analysis

From visual inspection of the data, Kolmogorov–Smirnov and Levene's test secured basic assumptions underlying parametric statistics. Level of protein carbonyls and amount yolk data (%) were logged and arcsine transformed, respectively. When analyzing rate of oxygen consumption, the data were split in two by a separate analysis of the 5°C-group to retain the assumption of linearity, which was compromised when analyzed together. In the case of cardiac performance, only eggs with beating hearts were included in the analysis. The effect of temperature and pH on development rate was analyzed by two-factor ANOVA. Before analyzing mean heart rate, rate of oxygen consumption, and level of protein carbonyls, the dependent measurements of each true replicate (clutch) were averaged, generating two independent and connected values per clutch, that is, one for the response variable and one for its corresponding yolk percentage. Thereafter, statistical analyses were performed by ANCOVA with% yolk as a covariate. Temperature effects were analyzed at different% yolk, depending on the fitting of the ANCOVAs, that is, 50% for heart rate, 37% for rate of oxygen consumption, and 55% for oxidative stress. Temperature and pH were treated as categorical factors. Significant effects were further investigated by post hoc analysis (Bonferroni corrections). Statistical analysis was carried out in SPSS Statistics (version 21 for MacOSX, Chicago, IL).

Development rate data were also analyzed with an Arrhenius plot in order to visualize a possible thermal break point. This inferred plotting the natural logarithm of the development rate on the *y*-axis and the inverse temperature on the *x*-axis. Arrhenius plots are frequently applied to the temperature behavior of complex biological systems (Clarke 1983). Furthermore, thermal sensitivity of development rate, heart rate, and rate of oxygen consumption were assessed by calculating *Q*_10_-coefficients with the Van't Hoff equation:


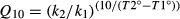


where *k*_1_ and *k*_2_ represent the rates at temperatures *T*_1_ and *T*_2_.

*Q*_10_ is a measure of the change in rate of a process with a 10°C change in temperature (Cossins and Bowler 1987), where a *Q*_10_ of 1 indicates no change. In biological systems, *Q*_10_-coefficients are usually between 2 and 3 (Clarke 1983).

## Results

### Experimental temperature and chemistry

Temperature did not deviate more than ± 0.01–0.07°C (SE) from aimed treatment. pH in the controls deviated by no more than ± 0.01 units, and for the low pH-group, a slightly larger fluctuation was measured, ± 0.02–0.03 units (Table 1).

**Table 1:** Experimental water chemistry data, measured 54 times per tempxpH treatment. Data are presented as mean ± SE. Pco_2_ values (mean ± SE) were calculated by CO_2_sys software (Pierrot et al. 2006). Dissociation constants from Mehrbach et al.*,* (1973), refitted by Dickson and Millero, 1987. Salinity of incoming seawater during the experiment was 32.0 ± 0.04 (mean ± SE) PSU

Temperature, °C		pH_total scale_^in situ^		TAμmol kg^−1^		Pco_2(μatm)_^in situ^	
Control	Low pH	Control	Low pH	Control	Low pH	Control	Low pH
5.16 ± 0.07	5.10 ± 0.07	8.11 ± 0.01	7.71 ± 0.02	2197 ± 17	2158 ± 25	330 ± 7	886 ± 31
10.00 ± 0.01	10.02 ± 0.01	8.02 ± 0.01	7.64 ± 0.02	2211 ± 19	2181 ± 27	422 ± 7	1096 ± 45
11.71 ± 0.02	11.71 ± 0.02	7.99 ± 0.01	7.59 ± 0.01	2255 ± 30	2229 ± 17	466 ± 9	1279 ± 44
13.57 ± 0.05	13.62 ± 0.06	7.95 ± 0.01	7.55 ± 0.02	2241 ± 24	2233 ± 22	518 ± 9	1414 ± 50
15.81 ± 0.03	15.72 ± 0.03	7.91 ± 0.01	7.53 ± 0.03	2262 ± 26	2233 ± 21	571 ± 9	1516 ± 59
17.87 ± 0.02	17.88 ± 0.05	7.87 ± 0.01	7.47 ± 0.02	2198 ± 19	2237 ± 17	632 ± 10	1787 ± 65

### Development rate of *Nephrops norvegicus* embryos

**Figure 1:**
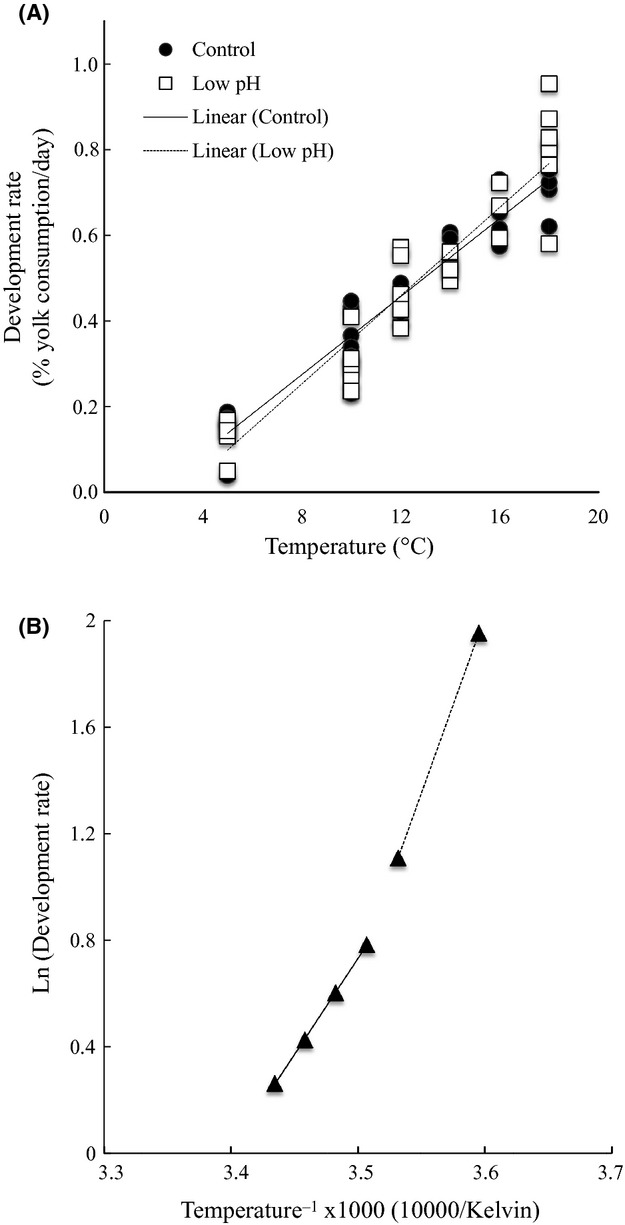
(A) Development rate in *Nephrops norvegicus* embryos, measured as yolk consumption (% yolk consumption clutch^−1^ day^−1^) in 72 clutches exposed to six different temperatures and 2 pH levels). Each data point represents one clutch. (B) Arrhenius plot of the average development rate, including both pH-levels (ln% yolk consumption clutch^−1^ day^−1^) versus incubation temperature (Kelvin^−1^ × 1000).

The rate of yolk consumption per day, as a measure of embryonic development rate, significantly increased with temperature (*P* < 0.001; Table 2) from 0.14 ± 0.01 at 5°C to 0.77 ± 0.03 (mean ± SE) at 18°C (Fig. 1A). Lower pH, however, had no effect on development rate (*P* = 0.86; Table 2) with an average rate of 0.48% yolk d^−1^ for both treatment groups (Fig. 1A). The Arrhenius plot revealed a thermal break point between 5–10°C and 10–18°C (Fig. 1B). The *Q*_10_-coefficient of 5–10°C was 2.5 units higher than the *Q*_10_-coefficient of 10–18°C, 5.4 versus 2.9.

**Table 2:** Results from the statistical analyses. Statistically significant *P*-values are presented in bold

Variable	Source	df	*F*	*P*-value	Statistics
Development rate	Temp	5	122	**0.000**	ANOVA
	pH	1	0	0.862	
	Temp × pH	5	1	0.278	
	Error	51			
Heart rate	Temp	5	12	**0.000**	ANCOVA
	pH	1	0	0.815	
	Temp × pH	5	1	0.611	
	% yolk	1	24	**0.000**	
	Error	46			
Oxygen consumption	Temp	4	5	**0.003**	ANCOVA
	pH	1	4	**0.051**	
	Temp × pH	4	1	0.727	
	% yolk	1	32	**0.000**	
	Error	41			
Oxidative stress	Temp	5	2	0.098	ANCOVA
	pH	1	8	**0.008**	
	Temp × pH	5	2	0.164	
	% yolk	1	32	**0.000**	
	Error	50			

### Cardiac performance of *Nephrops norvegicus* embryos

**Figure 2:**
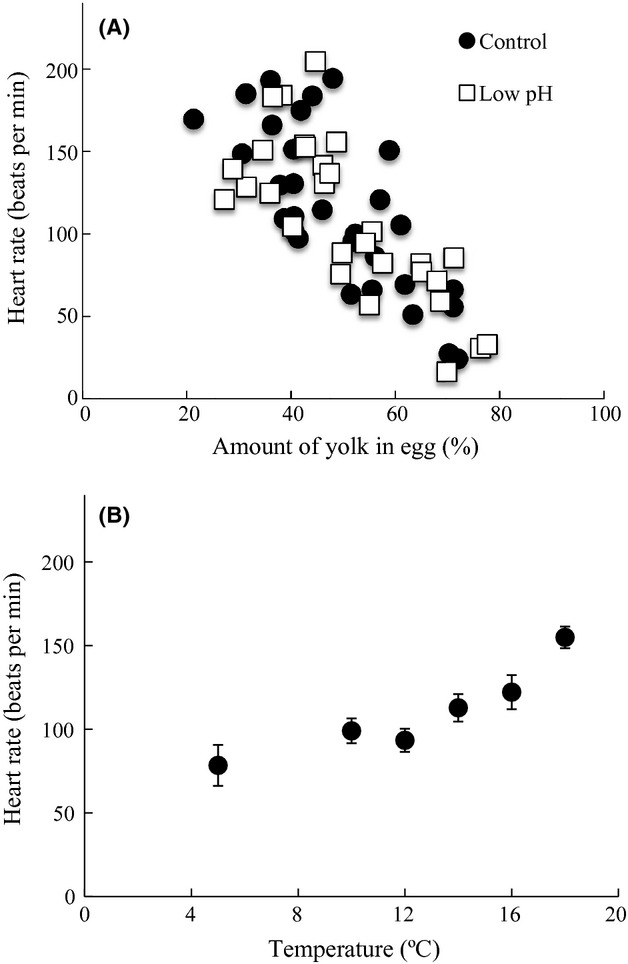
Heart rate in *Nephrops norvegicus* embryos (average beats min^−1^ clutch^−1^) in relation to (A) amount of yolk (average% yolk clutch^−1^) for 67 included clutches, divided into the two pH-treatments, low pH and control, and (B) temperature at a standardized developmental stage of 50% yolk (estimated by ANCOVA) including both pH-levels and independent of development (error bars SE, *n* = 7–12).

Mean heart rate increased significantly as the amount of yolk decreased (*P* < 0.001, Table 2) from 58 BPM at 70% yolk to 166 BPM at 30% yolk (Fig. 2A). There was also a significant increase in mean heart rate by temperature (*P* < 0.001, Table 2) from 78 ± 12 BPM at 5°C to 155 ± 6 BPM at 18°C (mean ± SE) (Fig. 2B). The *Q*_10_-coefficient increased from 1.3 between 5 and 12°C to 2.3 between 12 and 18°C. The pH had no effect on heart rate (*P* = 0.82; Table 2), and there was no interaction between pH and temperature (*P* > 0.61; Table 2) (Fig. 2A).

### Rate of oxygen consumption of *Nephrops norvegicus* embryos

**Figure 3:**
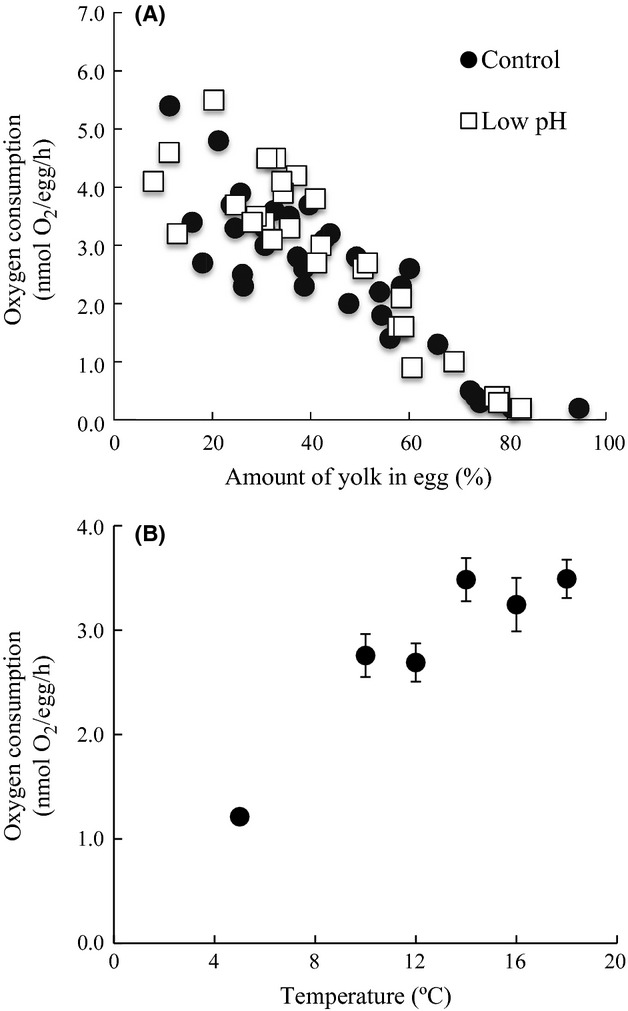
Rate of oxygen consumption rate in *Nephrops norvegicus* embryos (average nmol O_2_ egg^−1^ h^−1^) in relation to (A) amount of yolk (average% yolk clutch^−1^), for 52 included clutches, divided into the two pH-treatments, low pH and control, and (B) temperature at a standardized developmental stage of 37% yolk (estimated by ANCOVA), including both pH-levels (error bars SE, *n* = 8–12).

Rate of oxygen consumption increased significantly as the amount of yolk decreased (*P* < 0.001; Table 2) from 1.42 at 70% yolk to 3.47 at 30% yolk (Fig. 3A). It also increased with elevated temperature, independent of embryonic age (*P* = 0.003; Table 2). However, rate of oxygen consumption appeared to level out between 14 and 18°C (Fig. 3B). The *Q*_10_-coefficient decreased from 5.2 between 5 and 10°C to 1.8 between 10 and 14°C and finally to 1 between 14 and 18°C. Although not statistically significant (*P* = 0.051; Table 2; Fig. 3A), rate of oxygen consumption in the low pH-treatment was slightly higher than in the control pH. No interaction between pH and temperature was found (*P* = 0.73; Table 2). As the 5°C-group was not included in the ANCOVA that value was instead estimated later from the linear equation of the 5°C-group at the same amount yolk, that is 37% (Fig. 3B).

### Oxidative stress

**Figure 4:**
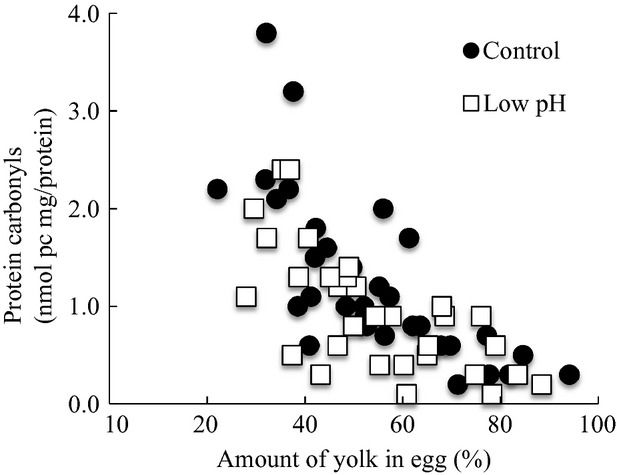
Oxidative stress in *Nephrops norvegicus* embryos, measured as concentration of protein carbonyls (average nmol pc mg protein^−1^) in relation to amount of yolk (mean% yolk clutch^−1^) for 68 included clutches divided into the two pH-treatments, low pH and control.

Oxidative stress measured as level of protein carbonyls increased significantly as the amount of yolk decreased (*P* < 0.001; Table 2) from 0.60 at 70% yolk to 2.02 at 30% yolk (Fig. 4). The level of oxidative stress was also significantly higher in the control group than in the low pH-group (*P* = 0.008; Table 2; Fig. 4). There was no significant effect of temperature on oxidative stress when analyzed independent of embryonic age (*P* = 0.098; Table 2), and no interaction between pH and temperature was found (*P* = 0.16; Table 2).

## Discussion

In this study, we investigated the combined effects of pH and temperature on embryonic development and physiology, of the ecological and commercially important benthic crustacean, *N. norvegicus*. Our objective was to evaluate crustacean embryonic tolerance, based on a long-term experiment (4 months) with treatment levels, realistic for a near future. We found no interactions between the two stressors and no effect of pH on development rate, mean heart rate, or rate of oxygen consumption. The only significant effect of pH was on the level of oxidative stress, with a higher concentration of protein carbonyls in the control embryos. At standardized amount of yolk (i.e., embryonic age), elevated temperature increased development rate, mean heart rate, and rate of oxygen consumption but did not affect oxidative stress.

The insensitivity of development rate of *N. norvegicus* embryos to OA is in line with previous studies on early development in a range of crustaceans, for example, *Acartia tsuensis, Homarus gammarus*, *Echinogammarus marinus,* and *Petrolisthes cinctipes* (Kurihara and Ishimatsu 2008; Arnold et al. 2009; Egilsdottir et al. 2009; Ceballos-Osuna et al. 2013). However, low pH has been found to reduce development rate in several other crustacean species, for example, *Semibalanus balanoides, Hyas araneus,* and *Homarus americanus* (Findlay et al. 2009; Walther et al. 2010; Keppel et al. 2012). Development is also affected by reduced pH in many other marine invertebrates, including corals, sea stars, and mussels (Fabry et al. 2008). In the face of environmental stress, some processes, such as development, can be undisturbed at the expense of other processes, such as mineralization. If there are associated costs with maintaining embryonic development rate in *N. norvegicus* embryos, these costs would affect processes other than mineralization, because calcification is not enhanced until the final pelagic larval stage (Spicer and Eriksson 2003).

Temperature is a major controller of ectothermic development with higher temperatures leading to a shorter development time (Gillooly et al. 2002). Farmer (1974) suggested that the increase in development rate stabilizes above 15°C. However, our study supports no such limit as development rate increased linearly up to the highest temperature tested, 18°C. At 5°C, development proceeded, although at a slow rate. This contradicts earlier literature, where it has been suggested that development may arrest for up to 7 months at winter temperatures (Farmer 1974). A thermal break point was found at 10°C, with an increase in *Q*_10_ by almost twofold between the ranges of 10–18°C and 5–10°C, (*Q*_10_: 2.9 vs. 5.4). However, the actual break point might be found below 10°C as our study does not provide data for any temperature between 5 and 10°C. Thermal break points indicate a major change in temperature-dependent processes, and according to Hochachka (1991), *Q*_10_-values in the order of 2 suggest enzyme-based reaction rates. In the planktotrophic asteroids of Hoegh-Guldberg and Pearse (1995), the average *Q*_10_-value was high (12.6) close to their lower end of tolerance and low (2.0) at their upper end of tolerance. High *Q*_10_-values are common at very low temperatures for many metabolic processes and might indicate depression (Hochachka and Guppy 1987). The broad tolerance seen in *N. norvegicus* is in strong contrast to the situation described for some species of fish, which tolerate or prefer a considerable more narrow temperature span (Righton et al. 2010; Neuheimer et al. 2011).

In contrast to previous studies on embryos, for example, *Littorina obtusata* and *Petrolisthes cinctipes,* which both showed depressed heart rate under elevated Pco_2_, we found no effect of Pco_2_ on heart rate (Ellis et al. 2009; Ceballos-Osuna et al. 2013). We found heart rate to increase with elevated temperature whereas rate of oxygen consumption increased then reached a limit at 14°C. Frederich and Pörtner (2000) observed the same pattern in the adult spider crab, *Maja squinado,* that is, an increasing heart rate with temperature above the level where ventilation no longer increased. Moreover, the *Q*_10_-value of embryonic heart rate between 5 and 12°C was 1.3. This is in accordance with the *Q*_10_ of 1.25 found in adult *H. araneus* between 6 and 12°C and in developing *Artemia fransiscana between* 22 and 34°C (Spicer 1994; Walther et al. 2009).

We found an initial low level of oxidative stress, in eggs with close to a 100% yolk. This is in accordance with earlier finding where eggs seemed to be actively cleared from metabolic rest products, so that new generations started physiologically young (Hernebring et al. 2006; Fredriksson et al. 2012). Closer to hatching, oxidative stress was higher, probably due to the increase in metabolic rate causing a mismatch between oxygen demand and delivery (Monaghan et al. 2009). Although protein carbonyl levels have not been measured in crayfish embryos previously, we anticipate that the increased oxidative stress with development is normal. Oxidative stress is known to increase by environmental perturbation (e.g., Abele et al. 1998). Unexpectedly, in our study, there was a significantly higher level of oxidative stress in the control embryos compared with the embryos developed in low pH (−0.4 units). In developmental studies, effects of treatments are often difficult to disentangle from “normal” changes, due to development it self. However, the difference in oxidative stress is unlikely explained by developmental changes as there were no differences in development rate between the groups, and the average percentage yolk was almost the same, that is, 54.2 (control) and 54.6 (low pH). Moreover, our finding contradicts those results found in hepatopancreas of the adults*,* where oxidative stress was higher in the low pH-treatment (Hernroth et al. 2012). Matoo et al. (2013) investigated whether the negative impacts of elevated Pco_2_ and temperature previously found in bivalves could be explained by a persistent oxidative stress signal but found no support for this although previous studies have suggested this (Tomanek et al. 2011). The link between oxidative stress and pH is thus not straightforward. As oxidative stress was measured using different methods in *N. norvegicus* embryos (this study, protein carbonyls) and adults (Hernroth et al. 2012, advanced glycation end products), the potential effect of method choice needs further examination. While we have no mechanistic explanation for why the controls in our study had a higher level of oxidative stress, the data are in line with the overall pattern observed that lower pH has no negative impact on *N. norvegicus* embryos.

One plausible explanation to why elevated Pco_2_ and thus lowered pH had no negative effects in our study could regard adaptation to their habitat. The embryos of *N. norvegicus* develop externally and during incubation the berried female tends to remain in her burrow. Although the pH in *N. norvegicus* burrows is not known, pH in burrows of smaller invertebrates, like polychaete worms are lower than in the overlying water (Zhu et al. 2006). This is due to the strong pH gradients in the sediment-near water interface. Exposing adults to elevated CO_2_ during reproductive conditioning may also have positive carry-over effects on their offspring, as seen in oysters (Parker et al. 2012). *Nephrops norvegicus* embryos, predisposed to hypoxia are also known to be more tolerant to later hypoxic events (Eriksson et al. 2006). In addition, it is possible that the internal environment of the eggs naturally experience high Pco_2_ and that they therefore are supplied with sufficient protective mechanisms, to maintain an outward flow of CO_2_ and to counteract acidosis (Hamdoun and Epel 2007; Dorey et al. 2013). Furthermore, maternal effects such as ventilation (by fanning with pleopods) could increase oxygen supply to the egg clutch and compensate for the potential metabolic stress of developing in low pH-water. However, the experimental water was fully aerated at all times.

As a final remark, we observed no mortality nor abnormalities. This is in agreement with earlier findings on the closely related *H. gammarus* were survival of zoea I-IV was not affected by exposure to 1200 ppm CO_2_ (2100 projection) (Arnold et al. 2009). However, brooding crustaceans are known to remove dead eggs, possibly to avoid infections (Factor 1995).

In conclusion, we found embryos of *N. norvegicus* tolerant to the prolonged exposure of the range of temperatures (5–18°C) and pH (−0.4 units) tested in this study, even though the natural population lives at a mean annual temperature of 10°C and rarely is exposed to the extreme values used in this study. Global warming would initially be beneficial for this species. However, in combination with the oxygen deficiency (hypoxia) frequently occurring in the bottom water during autumn, it is likely that developing benthic organisms will be affected negatively in the future (Baden et al. 1990; Eriksson 2000), as the elevated temperature will infer a higher metabolic rate. In addition, the hypoxic events co-occur with early embryonic development and have been reported to increase; in frequency, severity and areal due to anthropogenic activity and thermal expansion (Diaz and Rosenberg 2008).

We therefore anticipate that further studies on this species will combine elevated temperature, OA, and hypoxia. Whether an additional increase in temperature would speed development further without causing damage to the developmental program is also still to be tested.
